# Correction: Intellectual capital and the efficiency of SMEs in the transition economy China; Do financial resources strengthen the routes?

**DOI:** 10.1371/journal.pone.0240774

**Published:** 2020-10-13

**Authors:** Guowei Li, Zhe Luo, Muhammad Anwar, Yuqiu Lu, Xiantao Wang, Xuening Liu

The affiliation for the second author is incorrect. Zhe Luo is not affiliated with #1 but with #3: School of Public Administration, Sichuan University, Chengdu, China.
