# Supplementary material for: Intellectual capital and the efficiency of SMEs in the transition economy China; Do financial resources strengthen the routes?
Source: PLoS One. 2020 Jul 2;15(7):e0235462. doi: 10.1371/journal.pone.0235462 (PMC7332020; doi:10.1371/journal.pone.0235462)
Supplement: S1 Appendix — (DOCX) [file pone.0235462.s001.docx]

**Appendix**

The following questionnaire is used for data collection from Chinese SMEs. Owners/Managers were asked “*Please circle one of the following option relevant to your company”.*

*We deal Business of:* 1. Manufacturing 2. Trading 3. Services

*Number of Employees in our Company:*

1*.* 20 to 100 2. 101-200 3. 201-300, 4. 301-400, 5. 401-500

*Our firm operated since*

1. 10 years and less, 2. 11 and 20, 3. 21 above years

*My position in the company/firm/organization*…………………………………………………

*My education is:* 1. Intermediate and less 2. BA/B.Sc/B.com 3. MA/MBA 4. M.Phil 5.PhD

*Name of our Firms/Company/Organization. _______________________________________*

*Location / City in which company operates________________________________________*

*Circle the options based the given information.*

| Strongly Disagree | Disagree | Neutral | Agree | Strongly Agree |
| --- | --- | --- | --- | --- |
| 1 | 2 | 5 | 4 | 5 |

**Human Capital** Source: Liu [14]

1. We are active in upgrading employees' skills.
2. Our employees are bright.
3. Our employees are the best in industry.
4. Our employees are satisfied.
5. Our employees always come up with new ideas.

**Customer Capital** Source: Liu [14]

1. Our customers generally satisfied.
2. We reduce time needed to resolve customer or client problems.
3. Our organization provides value-added service to customers.
4. We are confident about the future with our customers.
5. We are maintaining longevity in our relationships with customers.
6. We receive lots of feedback from customers.

**Structural Capital** Source: Jain, Vyas and Roy [48].

1. Our firm is having a common system of values and beliefs directed towards company objectives
2. Our firms are having a well-documented systems and procedures
3. Our firm owns several patents, licenses and copyrights
4. Our firm uses CITs for communication, coordination, and information distribution

**Financial Resources** Memon, An and Memon [79].

1. We are satisfied with the financial capital available for the business operations
2. Our company has easy access to financial capital to support its business operations
3. Our business operations are better financed than our key competitors' operations
4. If we need more financial assistance for our business operations, we can easily obtain it
5. We have adequate financial resources for new product and internationalization process.

**SMEs efficiency** Memon, An and Memon [79], [9]

1. Return on equity
2. Return on sales
3. Return on investment
4. Return on assets
5. Sales growth
6. Net profitability
7. Market shares
8. Customers’ satisfaction
