# Supplementary material for: Intellectual capital and the efficiency of SMEs in the transition economy China; Do financial resources strengthen the routes?
Source: PLoS One. 2020 Jul 2;15(7):e0235462. doi: 10.1371/journal.pone.0235462 (PMC7332020; doi:10.1371/journal.pone.0235462)
Supplement: Attachment — Submitted filename: Response to Reviewers Comments.docx [file pone.0235462.s002.docx]

We are thankful to the reviewers and editor for the valuable comments on our manuscript. We have revised all the comments and have added worthy information to the research. We believe that the revised version is has much improved in terms of contents and validity.

**Review Comments to the Author**

Please use the space provided to explain your answers to the questions above. You may also include additional comments for the author, including concerns about dual publication, research ethics, or publication ethics. (Please upload your review as an attachment if it exceeds 20,000 characters)

Reviewer #1: The manuscript holds twofold novel contributions, include;
1. Testing the relationship between intellectual capital and SMEs efficiency that is perhaps missed in the previous studies. As the relationship between intellectual capital and SMEs performance is widely discussed but the efficiency of SMEs is very rarely studied in existing researches. This is important for ventures competing in the transition economy China.
2. The relationship is tested in the presence of financial resources that gives a plus point to the novelty. Indeed both intangible (intellectual capital) and tangible (financial resources) are very important for a venture growth.

Ans: Thank you for the positive comment.

However, I have a few minor concerns in this research that are highlighted below;
• I see that major parts are already covered in this introduction but the authors need to discuss the policy implications in detail.

Ans: Thank you for your comments. We have discussed policy implications in the introduction as shown in red color.
• Criteria for sampling approach is not well-explained, so it may be explained clearly.

Ans: Thank you. We have discussed it in detail.

• Procedure of the data collection need to be stated in an articulated way.

Ans: We have discussed it in detail, please read red color

• Authors need to discuss why they control the particular variables.

Ans: We have added a new section where it is discussed why we use control variables.

• Please provide a short note under the table 4, so that the readers can understand the idea.

Ans: Thank you for your comments. We have written a short note below table 4.
• In the discussion chapter, I suggest to provide a brief summary of major findings because it is very important in social science studies.

Ans: Thank you, we have provided a short summary of major findings, please read red color text.
Additionally, several grammatical and English structural mistakes are found in this manuscript, that need to be corrected via professional proofreading.

Ans: Thank you. We have proofread the manuscript from an English expert that can give clear description now.
The rest of the work is impressive, and the manuscript is recommended for possible acceptance after the minor correction are made accordingly.
Good luck.

**Reviewer #2: Thanks for the opportunity to revise this paper.**

This work explores the role of financial resources in firm efficiency as well as how financial resources strengthen the relation between IC and SMEs efficiency. I like the gap of the paper as well as the structure, development, and especially I think it contains good contributions, conclusions and implications. However, there are several changes that I have to suggest, in order to correct this version of the paper, because some of these contributions are questionable according with both theoretical and empirical questions.

Please, I invite the authors to reflect and modify the paper in the next questions.

INTRODUCTION:
The authors should emphaticise the importance of connecting IC dimensions’ to efficiency and competitive advantage and they should show more clearly, if they focus on efficiency or competitive advantage and why.

Ans: Thank you. We have added a new paragraph in the introduction where the paths between the dimensions of IC and SMEs success have been stated.

They should introduce more clearly, they chose resource base view and no other organizational theories for their research.

Ans: We have shed light on the RBV theory as it underlines our conceptual model because it convers tangible (financial resources) and intangible resources (IC). Moreover, several other studies have discussed the RBV theory when they tested the path between IC and SMEs performance.

Khan, S. Z., Yang, Q., & Waheed, A. (2019). Investment in intangible resources and capabilities spurs sustainable competitive advantage and firm performance. *Corporate Social Responsibility and Environmental Management*, *26*(2), 285-295.

Anwar, M., Khan, S. Z., & Khan, N. U. (2018). Intellectual capital, entrepreneurial strategy and new ventures performance: Mediating role of competitive advantage. *Business and Economic Review*, *10*(1), 63-93.

THEORETICAL BACKGROUND AND HYPOTHESES DEVELOPMENT
Please if you are going to use efficiency as a central key……..do not use it collaterally (eg. Pg 10). I advise you to introduce one or two paragraphs that explain the importance of efficiency in general and specifically in SMEs.

Ans: Thank you for the useful suggestion. We have added a new paragraph stating three types of efficiency and how these are importance in SMEs sector, please read under the literature section.

Please, I’d like to read how the authors use Resource based View with great depth, for example for explaining how IC includes or not the traditional requirements (VRIO) of this theory for achieve competitive advantage. It can help a lot to strengthen the H1, H2 and H3. In this version, the arguments are too general and without using the basic concepts of the theory.
Please, use more clearly the SMES’s characteristics for developing the hypothesis……….Certainly it could be one of the major contributions of the paper (SMEs context).

Ans: Thank you for your comments. We have discussed the theory in detail and how it is matched our model and why SMEs need IC are discussed in the literature section, please read red text.

Regarding the moderated role of financial resources, the arguments are more clear and robust.

Ans: Thank you.

METHODOLOGY:
According the representativeness of the simple, how do you check the difference between the response and non-response firms?

Ans: Thank you for pointing out this issue. We have executed a new test to check the problem that have discussed. We are aware which firm provided early response and which is late, because as we received our information, we are continuously entered in the SPSS file.

Are you sure it is good idea to measure firm efficiency with self-reported answers of owners/managers?

Ans: We appreciate the point raised by the reviewer. Indeed we have faced this question many time in top ranked journals. We have discussed in the manuscript why self-reported measures are better in case of SMEs. Please read the measurement section and particularly “efficiency”

How do you manage the social desirability bias? Why not do you use some other secondary variable to check unless efficiency?

Ans: Thank you for the comment. We have mentioned why efficiency is self-reported measured and have provided evidence based reasons under the measurement section. Please read the red part.

You say: “GFI = 0.81, CFI = 0.87, TLI = 0.85 and NFI = 0.81 displayed acceptable values (closed to 0.90)” ……..Are you sure? I am not. These values are so lower for saying that they are acceptable values.

Ans: The reviewer has raised a valid point. There is no universal criteria for a model fit. However, values greater than 0.80 are considered acceptable when CMIN/DF is less than 3.

The values greater than 0.80 are mentioned in the following studies;

- Wales, W. J., Patel, P. C., & Lumpkin, G. T. (2013). In pursuit of greatness: CEO narcissism, entrepreneurial orientation, and firm performance variance. *Journal of Management Studies*, *50*(6), 1041-1069.
- Panigyrakis, G. G., & Theodoridis, P. K. (2007). Market orientation and performance: An empirical investigation in the retail industry in Greece. *Journal of Retailing and Consumer Services*, *14*(2), 137-149.
- Liu, C. H. (2017). The relationships among intellectual capital, social capital, and performance-The moderating role of business ties and environmental uncertainty. *Tourism Management*, *61*, 553-561.

According validity and reliability of the variables, I see that in each variable there is some item that has a very low value (see table 4). Hc4 in Human Capital, sc2 in Structural Capital cc3 in Customer capital, fr4 in financial resources or fe7 in SMEs Efficiency.

Ans: Again a valid question. However, I would like to discuss it for clarity. Hair et al., (2006-2010) stated that an item with standard factor loading above 0.70 should be retained and below 0.40 should be dropped. However, items in range of 0.41-0.69 should be retained or dropped after calculating their average. For instance, if there are three items with 0.80, 0.50 and 0.90 loading respectively. The average value of these three factor is 0.70, hence the item with a lower factor loading (0.50) can be retained. On the basis of this suggestion, we have retained the items. The same approach is followed in several studies such as

Anwar, M., Rehman, A. U., & Shah, S. Z. A. (2018). Networking and new venture’s performance: Mediating role of competitive advantage. *International Journal of Emerging Markets*.

Khan, S. Z., Yang, Q., & Waheed, A. (2019). Investment in intangible resources and capabilities spurs sustainable competitive advantage and firm performance. *Corporate Social Responsibility and Environmental Management*, *26*(2), 285-295.

How do you use control variables (size and age), as a categorical variable or continuous variable? If first, I think it is wrong to lose the information of having the continuous data.

Ans: Thank for the worthy comment. We have discussed the control variables in detail. Please read the methodology section where it is stated that nature of industry is a categorical variable and size and age of firms as well as educational background are continuous variables that are controlled directly in structural model.


In regards to Figure 4,5,6……..which is p-value for the Gradient slope? It is not sufficient with showing the image.

Ans: Thank you. We have discussed the p values, highlighted red.

In summary, this very good potential paper needs important improvements. The gap and the sample is appropriate, but both theoretical and empirical changes need to be done.

Ans: Thank you for the comment. We have discussed theoretical section in detail and have applied regression analysis in SPSS to check if there is difference in the results.

I encourage the authors to develop these changes in order to improve the paper. Good luck!

Thank you once again for your time and consideration.
