# Supplementary material for: Intellectual capital and the efficiency of SMEs in the transition economy China; Do financial resources strengthen the routes?
Source: PLoS One. 2020 Jul 2;15(7):e0235462. doi: 10.1371/journal.pone.0235462 (PMC7332020; doi:10.1371/journal.pone.0235462)
Supplement: Attachment — Submitted filename: Response to Reviewers Comments.docx [file pone.0235462.s003.docx]

We are thankful to the reviewers and editor for the valuable comments on our manuscript. We have revised all the comments and have added worthy information to the research. We believe that the revised version contains worthy implications and is ready for publication.

**Reviewer #2: All comments have been addressed**

All the comments are addressed.

Ans: Thank you very much for the time and suggestions of the reviewer that enabled us in building a quality research work.

Reviewer #1:

I feel that most of the recommendations have been well conducted. However, there are 2 issues that I consider have not yet been resolved by the authors. These two questions are relevant, especially the second, and their resolution would increase the robustness of the results of the work.

These questions are the following:
First, I still disagree on the use of size and age variables as categorical and not as continuous variables. I believe that this issue remains unresolved and I suppose I think this question can be solved, probably with a simple phone call to the company, for example. The use of these variables as categorical reduce their explanatory power of the model even more in a sample that is already SMES.

Ans: We appreciate the point raised by the reviewer to reduce spurious results and increase explanatory power of the model.

However, it is a challenge for us to know when the venture is started and how many people are exactly working in the firms. Because the survey is conducted using a hard copy approach before the COVID-19 crises and it was easy for us to go and connect with responsible managers. Since the crises have changed the situation and enterprises have not yet formally started their operational activities physically but they are working virtually. We have tried to contact the enterprises who have participated in the survey but contacting via call did not favor. We have called several firms, in most of the cases, the bottom level managers are connected and excused to talk top management team. Unfortunately, response of the bottom level managers was not satisfactory for us because only top managers and owners understand strategic policies, performance activities and decision making process of their firms (Degong et al., 2018; Songling et al., 2018; Tajeddini and Milluer, 2012).

We realized that it is big challenge and perhaps impossible to reach and connect with all the enterprises (264) in Beijing, Shanghai and Shenzhen (e.g. due to the current situation-COVID-19) who have already provided their responses via a hard copy questionnaire.

Additionally, we have read a few studies in similar nature who have also used the same strategy for age and size of the enterprises.

1. Khan, S. Z., Yang, Q., Khan, N. U., Kherbachi, S., & Huemann, M. (2020). Sustainable social responsibility toward multiple stakeholders as a trump card for small and medium‐sized enterprise performance (evidence from China). *Corporate Social Responsibility and Environmental Management*.
2. Memon, A., Yong An, Z., & Memon, M. Q. (2020). Does financial availability sustain financial, innovative, and environmental performance? Relation via opportunity recognition. *Corporate Social Responsibility and Environmental Management*, *27*(2), 562-575.
3. Khan, S. Z., Yang, Q., & Waheed, A. (2019). Investment in intangible resources and capabilities spurs sustainable competitive advantage and firm performance. *Corporate Social Responsibility and Environmental Management*, *26*(2), 285-295.
4. Ilyas, S., Hu, Z., & Wiwattanakornwong, K. (2020). Unleashing the role of top management and government support in green supply chain management and sustainable development goals. *Environmental Science and Pollution Research*, 1-14.
5. Anwar, M. (2018). Business model innovation and SMEs performance—Does competitive advantage mediate?. *International Journal of Innovation Management*, *22*(07), 1850057.

Second, I recognize that there are papers where efficiency can be measured with self-reported methods. But it is also evident that the limitation of work is then very important especially if other control measures are not taken and there is clearly an effect of social desirability bias. There are other steps that can be taken to address this issue, such as surveying performance issues from a different source (for example, other person).

Ans: Thank you once again for raising the valid comment of social desirability bias in performance measures. The reviewer is right that there are papers that have measured the efficiency as a self-reported method. This is especially true in case of SMEs because they do not provide their financial information to the general public, as stated in the manuscript. Also this claim is stated in several research papers measuring efficiency:

1. Khattak. M.S. Shah. S.Z.Z (2020). Top Management Capabilities and Firm Efficiency: Relationship via Resources Acquisition. *Business and Economic Review,* 12(1), 87-118. <https://imsciences.edu.pk/files/journals/vol12_2020/New%205-%20667.pdf>
2. Martin, S. L., & Javalgi, R. R. G. (2016). Entrepreneurial orientation, marketing capabilities and performance: the moderating role of competitive intensity on Latin American International new ventures. *Journal of Business Research*, *69*(6), 2040-2051.

To avoid the problem, we have already applied a few measures; validity, reliability and common method variance that are recommended in other studies, listed above. Additionally, we requested owners and top managers as they are the people who know the financial position and strategic activities of their firms. Additionally, where managers were not aware of exact financial figures, they recommended us to ask financial managers regarding efficiency and financial outcomes. Not in all the firms but in most cases, the survey is not filled by a single person, especially in those enterprises where agents were working of the behalf of owners of the firms.

We asked them that the survey is volunteer to be filled. To reduce social desirability bias, we have clearly mentioned in the covered letter of the survey that the data of this survey is exclusively used for research analysis, and the information will not be shared elsewhere.

Additionally, we read a few papers where they have used open ended questions/interview as a robustness check for mitigating social desirability bias and improving validity of the results.

- Anwar, M., Khan, S. Z., & Khan, N. U. (2018). Intellectual capital, entrepreneurial strategy and new ventures performance: Mediating role of competitive advantage. *Business and Economic Review*, *10*(1), 63-93.
- Saad, A., Xinping, G., & Ijaz, M. (2019). China-Pakistan Economic Corridor and its influence on perceived economic and social goals: Implications for social policy makers. *Sustainability*, *11*(18), 4949.

Perhaps this was a suitable method for us to follow this approach. Parsimoniously, we relied on this approach and conducted a brief interview with five firms that has discussed below.

**Online interviews**

We conducted an in-depth interview with five managers/owners of the SMEs to enhance validity of the results and especially to know if there is social desirability bias in the data. We asked the following questions in the interview to get open ended answers.

1. Elaborate your IC strength in terms of human capital, structural capital and customers’ capital and how it benefits your firms, especially performance and efficiency?

Ans: Three managers shed light on the importance of their IC for high performance and efficiency while two firms replied that their IC averagely contribute to their performance.

2. How your existing financial resources moderate the path between IC and efficiency?

Ans: Four managers said that they get high benefits of IC when financial resources work as a moderator while one manager said that my firm faces financial constraint, and we are unable to get desirable efficiency.

3. Generally, do you agree that your performance and efficiency depend on IC and financial resources?

Ans: Three managers were agreed and said that IC and financial resources are the key to performance and efficiency in the current competitive markets. However, two managers said that other types of resources; IT, online business activities and reputation.

To summarize the interview results, we hereby confirm that overall, the results match with the survey data where the dimensions of IC and financial resources are considered crucial factors of high efficiency and performance.

I encourage authors to make one last effort to resolve these issues, especially the issue of social desirability bias.

Once again, we appreciate the points raised by the reviewer and advancing our knowledge to mitigate this issue in future studies.

Additionally, the reviewer is also welcome if she/he still sees elusiveness in the results. As realized in the online survey during the COVID-19, we will have only option to collect new data from random enterprises that may or not may include the firms who have participated in the structured survey via a hard copy approach. In this case, we have to analyze the results again because of new data, and see what are the new results are, perhaps similar or different. So we can take time to conduct the online survey due to COVID-19 in order to complete the analysis in a new way.

We hope that the reviewer will agree and favor our comment. Thank you once again for your time and comments that enabled us in building a wonderful piece of research.
